# Sex differences in the pathogenesis of degenerative knee osteoarthritis: a comparative transcriptomics analysis

**DOI:** 10.3389/fragi.2026.1875372

**Published:** 2026-08-06

**Authors:** Ayobami S. Ogunsola, Davis J. Brady, Jeffrey A. Foster, Marcel G. Brown, Molly A. Hartzler, Maxwell K. Langfitt, John S. Shields, Xue Ma

**Affiliations:** 1 Atrium Health Wake Forest Baptist, Department of Orthopaedic Surgery and Rehabilitation, Winston-Salem, NC, United States; 2 Wake Forest University School of Medicine, Winston-Salem, NC, United States

**Keywords:** cartilage, differentially expressed genes (DEGs), functional enrichment analyses, osteoarthritis, primary osteoarthritis of the knee, RNA sequencing, sex differences, transcriptomics

## Abstract

**Introduction:**

Osteoarthritis (OA) is one of the leading diseases worldwide and is expected to continue to rise in prevalence. Epidemiological studies on OA have long shown a sex discrepancy between males and females, with females consistently showing greater rates of OA. There are numerous theories about why this might be the case; however, the discrepancy is still poorly understood.

**Methods:**

In this study, two cohorts of patients with Kellgren-Lawrence grade IV knee OA were grouped based on sex and analyzed to decipher transcriptomic differences. The first cohort was a GSE114007 public dataset from the NCBI Gene Expression Omnibus (GEO) database, while the second cohort was from our institution. For each cohort, differential gene expression analysis (DEG), principal component analysis (PCA), functional enrichment analyses (Kyoto Encyclopedia of Genes and Genomes, Gene Set Enrichment Analysis, and Gene Ontology), and protein-protein interaction (PPI) analysis were performed.

**Results:**

In the GSE114007 cohort, XIST gene was the sole significant DEG at the genome-wide level. In contrast, our local cohort showed 109 DEGs between males and females. Functional enrichment analyses in both the GSE114007 and local cohorts showed female upregulation of inflammatory pathways, while the local cohort also showed female increases in protein synthesis and metabolic pathways, with males showing greater enrichment of structural and tissue remodeling pathways. PPI analysis showed female upregulation of three clusters of interacting proteins related to protein synthesis and translation, energy metabolism, oxidative phosphorylation, cell communication, and tissue remodeling.

**Discussion:**

Our analyses suggest that the pathogenesis of OA in women may be related to inflammatory and metabolic mechanisms, whereas OA in men may be driven by structural pathways involved in stress and remodeling. Hence, sex specific therapeutic strategies may be necessary to effectively target the pathobiological mechanisms driving OA in males and females.

## 1 Introduction

Osteoarthritis (OA) is a chronic degenerative disease of the joint that often results in joint pain, disability, and overall decreased quality of life (Cui et al., 2020; Long et al., 2022; Yan et al., 2022). In the United States, the prevalence of OA ranks high among all diseases, with an estimated 33 million Americans affected (Centers for Disease Control and Prevention, 2025). Predictions suggest that global prevalence will continue to increase due to an increasingly aging population (Steinmetz et al., 2023). Epidemiological trends show a significantly higher prevalence of OA in female populations (up to 60%) compared to male (Ogunsola et al., 2024; Segal et al., 2024; World Health Organization, 2023).

Biological, anatomic, comorbidity/lifestyle, and genetic differences have been explored to explain this gender discrepancy in OA. However, to date, no definitive mechanisms have been established (Segal et al., 2024). Segal et al. recently published a comprehensive literature review of many factors that may contribute to differences in OA prevalence between males and females (Segal et al., 2024). Some of these contributing factors are briefly discussed below.

Hormonal differences are thought to play a role in the increased prevalence of OA in females. A significant decline in cartilage-protective estrogen during the postmenopausal period may be implicated in the increasing prevalence and progression of OA in females (Boyan et al., 2013a; Sniekers et al., 2008). Additionally, the cyclic nature of sex-dependent hormones in females has been shown to lead to greater laxity of the ligamentous structures. This can result in joint hypermobility, which can lead to early anterior cruciate ligament rupture, thereby increasing the risk of OA (Boyan et al., 2013b). Moreover, attributing the sex differences seen in OA to only the systemic estrogen effect may be overly simplistic, as factors including hormone mechanism of action and receptor numbers in chondrocytes may also drive sex-specific variations (Boyan et al., 2013b; Kinney et al., 2005). A clinical trial following 200 symptomatic patients (107 males and 93 females) with knee OA placed on vitamin D supplements measured the serum levels of sex hormones at baseline and 24 months, as well as knee magnetic resonance imaging (MRI) scans during the same period. It revealed that low levels of serum estradiol, progesterone, and testosterone are associated with increased knee effusion-synovitis in females, and higher levels of progesterone in females were correlated with higher cartilage volume (Jin et al., 2017).

Studies examining knee OA have shown that limb alignment is an important indicator of knee OA incidence (Nicolella et al., 2012; Segal et al., 2013). Generally, varus malalignment leads to increased medial compartment stress while valgus malalignment leads to lateral compartment stress. A systematic review and meta-analysis of coronal plane alignment of the knee (CPAK) distribution across knee phenotypes found that valgus malalignment (CPAK type III) was more common in European and Asian females with knee OA compared to males, while varus malalignment (CPAK type I) was more common in European males with knee OA compared to females (Giurazza et al., 2025). Similarly, Xin et al. found that females had a higher risk of developing lateral knee OA than males, with valgus malalignment accounting for 23% of this effect (Xin et al., 2024). These findings may be explained by females often having larger quadriceps (Q) angle, genu recurvatum, anterior pelvic tilt, and femoral anteversion, which are potential risk factors for injury and knee OA (Ekim et al., 2017; Faber et al., 2024; Mandal and Saha, 2024; Medina McKeon and Hertel, 2009). Moreover, males have been shown to have a greater amount of cartilage and more biomechanically favorable cartilage morphology than females (Di Martino et al., 2024; Kumar et al., 2015). Gait itself might also play a role in OA, with females with knee OA having lower first peak knee adduction and peak knee adduction compared to males (Yamagata et al., 2025).

Additionally, obesity is a known risk factor for OA due to mechanical stress and pro-inflammatory milieu (Herrero-Beaumont et al., 2019; Nedunchezhiyan et al., 2022). Although obesity rates do not differ significantly between males and females, there is a higher rate of severe obesity in females (Emmerich et al., 2024). The association between knee OA and obesity has been found to be stronger in females due to an increase in fat distribution (e.g., waist circumference and waist-to-hip ratio) and reduced cartilage volume (Faber et al., 2024; Cicuttini et al., 1999; W et al., 2016).

More recently, clinical and epidemiological studies have begun to investigate a molecular etiology for the observed sex differences in knee OA. Joint metabolism biomarkers, COMP and CTX-II, have been shown to be associated with knee OA in females (Hu et al., 2022; Kaur et al., 2025), while higher synovial fluid C5 levels have been associated with decreased synovial vascularization in males with late-stage knee OA (Sodhi et al., 2022). From a genetic perspective, CaMK4, BMP4, KDM6A, JMJD1C, PRKX, ZFX, and LAMTOR5 have been identified as hub genes of interest for OA development in females compared to males, demonstrating expression within the MAPK and Thyroid hormone signaling pathways (Xu et al., 2022; Feng et al., 2023). The expression of IL2RB and TRAPPC2 has also been shown to be higher in female patients with knee OA compared to males, while the expression of PTPN2 has been shown to be lower in females with knee OA (Xue et al., 2023). While these biomarkers and genetic targets are promising, lacking consensus in the literature stresses the need for larger, sex-balanced cohort studies to identify molecular mechanism(s) that may strongly contribute to sex differences observed in knee OA.

Given that the observed sex differences in knee OA burden are likely to be multifactorial, the presented study aimed to identify potential hub genes that may contribute to sex differences in knee OA pathophysiology. The objective of this study was to use next-generation sequencing (NGS) data from the Gene Expression Omnibus Database (GEO) and our own patient cohort to study these sex differences in advanced-stage OA cartilage tissues. We hypothesized that male and female OA cartilage would exhibit distinct transcriptomic profiles reflecting sex-specific pathobiological mechanisms.

## 2 Materials and methods

Two independent cohorts were included to strengthen our analysis and reproducibility of our findings. The public GEO dataset (GSE114007) provided a larger, independent transcriptomic dataset for initial sex-based pathway analysis, while the local institutional cohort provided RNA-seq data generated under controlled collection from a well-characterized patient population.

### 2.1 RNA sequencing and data preparation

RNA sequencing data from cartilage samples of patients with Kellgren-Lawrence (KL) grade 4 OA and control healthy subjects were obtained from the NCBI Gene Expression Omnibus (GEO) database (https://www.ncbi.nlm.nih.gov/geo/). Raw human gene expression count data, GSE114007, were generated using Illumina HiSeq 2000 and Illumina NextSeq 500 and extracted from the GEO database. GSE114007 contains 18 healthy samples (5 female; 13 male) and 20 OA samples (12 female; 8 male). Healthy control samples were not included in the sex-based comparative analyses presented in this study. Only the OA samples of the GSE114007 was used for our analysis (Table 1). The analysis was further extended using cartilage samples obtained from patients (4 males and 4 females all with KL grade 4 OA) who underwent total knee arthroplasty at our academic hospital (Table 2). Institutional review board approval was obtained for collecting the cartilage samples. Inclusion criteria were primary (Idiopathic) knee OA with age <75 years; patients were excluded if they had current smoking history, diabetes, cancer or other immunosuppressive diseases, or any history of meniscus pathology or knee ligament injury. RNA extraction was performed using TRIzol reagent (Invitrogen). Total RNA was used to prepare complementary DNA (cDNA) libraries using Illumina® TruSeq® Stranded Total RNA Library Prep Globin (Illumina Inc.). RNA Integrity Number (RIN) values for RNA samples were assessed using an Agilent TapeStation. Briefly, 750 ng of total RNA was ribosomal RNA (rRNA)-depleted, followed by enzymatic fragmentation, reverse-transcription, and double-stranded cDNA purification using AMPure XP magnetic beads. The cDNA was end-repaired, 3′-adenylated, with Illumina sequencing adaptors ligated onto the fragment ends, and the stranded libraries were pre-amplified by polymerase chain reaction (PCR). The library size distribution was validated, and the quality was inspected using an Agilent TapeStation. The quantity of each cDNA library was measured using Qubit 3.0 (Thermo Fisher, United States). The libraries were pooled and sequenced on the Illumina NextSeq 2000. Trimmomatic v0.33 was used for trimming and cleaning raw sequencing data to ensure high-quality reads. The cleaned reads were aligned to the reference genome GRCh37/hg19 using STAR v2.5.2a. Finally, gene-level quantification was performed using featureCounts v1.4.5, which counted the number of reads mapped to each gene.

**TABLE 1:** Metadata of RNA sequencing gene expression samples abstracted from GEO database (GSE 114007).

OA sample Id	Age	Sex	KL OA grade
OA_01	52	F	Grade 4
OA_02	57	F	Grade 4
OA_03	67	F	Grade 4
OA_04	60	M	Grade 4
OA_05	51	M	Grade 4
OA_06	67	M	Grade 4
OA_07	62	F	Grade 4
OA_08	69	F	Grade 4
OA_09	71	F	Grade 4
OA_10	71	F	Grade 4
OA_Cart_1_7	75	F	Grade 4
OA_Cart_10_9	68	M	Grade 4
OA_Cart_2_8	71	M	Grade 4
OA_Cart_3_9	82	F	Grade 4
OA_Cart_4_10	62	F	Grade 4
OA_Cart_5_5	69	F	Grade 4
OA_Cart_6_1	70	M	Grade 4
OA_Cart_7_2	68	F	Grade 4
OA_Cart_8_5	66	M	Grade 4
OA_Cart_9_6	66	M	Grade 4

**TABLE 2:** Metadata of RNA sequencing gene expression samples from local patient cohort.

OA sample Id	Age	Sex	BMI	KL OA grade
640C	66	F	37.36	Grade 4
645C	68	F	27.98	Grade 4
653C	66	F	34.01	Grade 4
658C	73	M	37.16	Grade 4
660C	69	M	26.83	Grade 4
667C	62	M	35.60	Grade 4
674C	59	M	31.40	Grade 4
679C	61	F	21.60	Grade 4

### 2.2 Statistical analysis

The GEO and local cohort raw counts were filtered to remove low expressed genes using a minimum count threshold of 10 and a minimum sample requirement of 3. Genes that did not meet these criteria were excluded from our analysis to ensure that the dataset focused on biologically relevant and sufficiently expressed genes. After filtering, the GEO and local cohort raw counts were 15,475 and 20,799 genes, respectively. Next, we applied DESeq2 size factor normalization to account for differences in sequencing depth across the samples and performed sex-based differential gene expression (DGE) analysis separately on the GEO and cartilage samples using males as reference (Love et al., 2014). The DGE analysis used a default adjusted p-value threshold of 0.1 to determine significant genes. After DGE, functional annotation and pathway enrichment analysis were performed on the differentially expressed genes (DEGs) followed by protein-protein interactions (PPIs). The PPIs were created using the interaction gene retrieval tool STRING v.11.5, and the results were imported into Cytoscape software (v. 3.10.3) for further analysis. All the data analyses in this study were performed using R version 4.4.1 (R Core Team, 2024).

## 3 Results

### 3.1 Analysis of GSE114007 dataset

#### 3.1.1 Differential gene expression analysis between male and female knee OA patients

**FIGURE 1:**
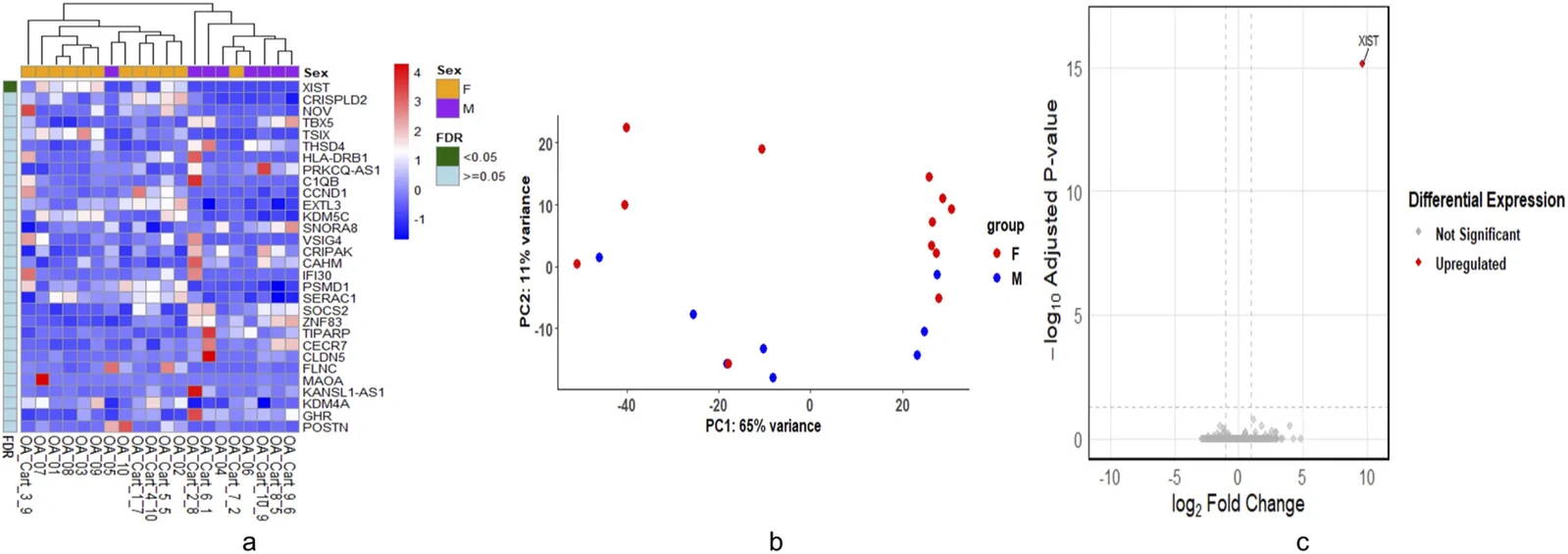
Differential gene expression analysis between male and female osteoarthritis patients. **(a)** Heatmap of the Top 30 Differentially expressed Genes (GSE114007). **(b)** Principal Component Analysis between male and female samples. **(c)** Volcano plot of differential gene expression analysis indicating that XIST was the only significantly upregulated gene in females.

From 15,475 genes with nonzero read counts, DGE analysis identified one upregulated gene (0.0065%) with an adjusted p-value of 0.1, whereas no downregulated genes were detected. The heatmap shows the top 30 differentially expressed genes between male and female patients with OA ranked by adjusted p-values. Samples were clustered based on sex, which suggests some degree of sex-specific expression patterns. However, the limited number of differentially expressed genes highlighted the subtlety of these differences (Figure 1a). Additionally, principal component analysis (PCA) was performed on variance-stabilizing transformed (VST) data to assess the overall clustering of samples based on global gene expression profiles. The PCA plot showed partial separation between sexes along PC1 (65% variance), though with some overlap, whereas PC2 (11% variance) may capture variability unrelated to sex (Figure 1b). This limited separation is consistent with the small number of significant DEGs identified in this cohort. The volcano plot indicated that XIST, a sex differentiating gene usually upregulated in females, was the only significantly upregulated gene, whereas most genes showed non-significant expression differences (Figure 1c).

#### 3.1.2 Functional annotation and pathway enrichment analysis

**FIGURE 2:**
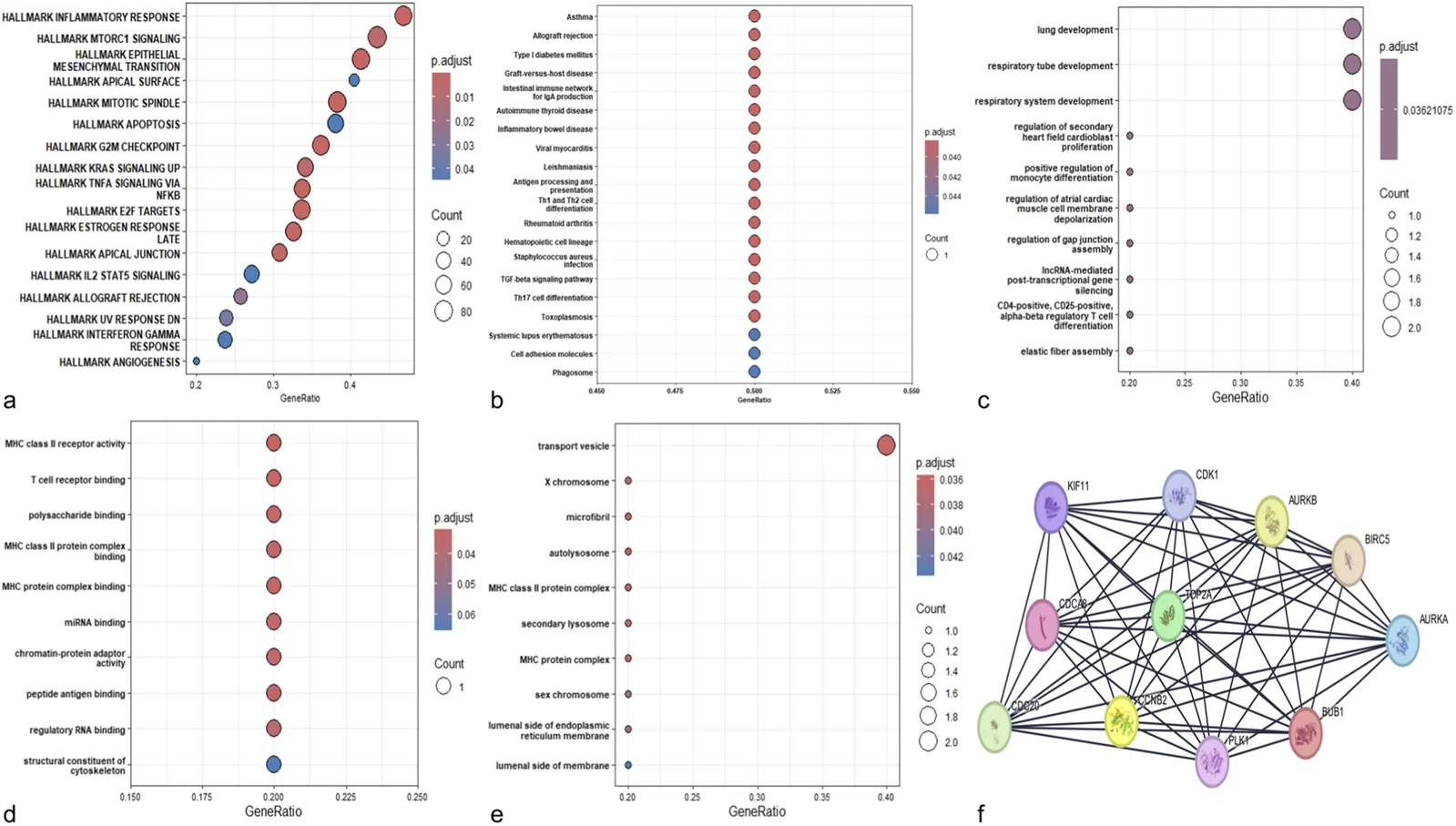
Functional enrichment analysis of DEGs between female and male osteoarthritis patients from GEO database. **(a)** GSEA identified hallmark pathways such as “inflammatory response” and “Hallmark TNF-α signaling via NF-kB (adjusted p-value <0.05). **(b)** KEGG pathway analysis showed inflammatory/immune-related pathways, such as “Th17 cell differentiation” and “Antigen processing and presentation” (adjusted p-value <0.5). **(c–e)** GO enrichment highlighted biological processes, molecular function, and cellular processes (adjusted p-value <0.5). **(f)** Protein-protein interaction (PPI) network showing highly interconnected subnetwork of key proteins, such as CDK1, AURKA, and TOP2A.

Kyoto Encyclopedia of Genes and Genomes (KEGG) pathway analysis, Gene Set Enrichment Analysis (GSEA), and Gene Ontology (GO) enrichment analyses were conducted to explore the possible biological mechanisms underlying sex differences in OA. Given the limited number of DEGs identified in the DESeq2 analysis, a lenient significance threshold was used (adjusted p-value <0.5) for KEGG and GO analyses to capture potential trends for exploratory purposes. These enrichment results should be interpreted as exploratory and hypothesis-generating, as several terms are supported by relatively few genes. GSEA was used to evaluate the enrichment of predefined gene sets across an entire ranked list of genes using log2 fold change. GSEA suggested significant enrichment of hallmark pathways related to inflammation, tissue remodeling, and cellular signaling in the GSE114007 gene expression data of females compared with male patients with OA (Figure 2a). Enriched pathways included hallmark inflammatory response, MTORC1 signaling, NF-kB signaling, and KRAS signaling pathways. KEGG pathway analysis showed a trend towards the enrichment of immune-related pathways, including asthma, rheumatoid arthritis, and systemic lupus erythematosus (Figure 2b). In addition, GO enrichment analysis identified the involvement of immune regulation, antigen presentation, and intracellular trafficking in OA, with enriched terms such as extracellular matrix organization, MHC class II activity, and T-cell receptor binding (Figures 2c–e). The protein-protein interaction (PPI) network was streamlined using a degree filter to ensure that only highly connected nodes were retained, in addition to using a high combined score (>900) to focus on meaningful interactions. The PPI network identified several hub proteins, including CDK1, PLK1, and TOP2A, that play key roles in cell cycle regulation and apoptosis (Figure 2f). Other identified hub proteins include AURKA, AURKB, BIRC5, BUB1, CCNB2, CDC20, CDCA8, and KIF11, which play central roles in mitotic regulation and cartilage remodeling.

### 3.2 Patient cohort analysis

#### 3.2.1 Differential gene expression analysis between male and female knee OA patients from local cohort

**FIGURE 3:**
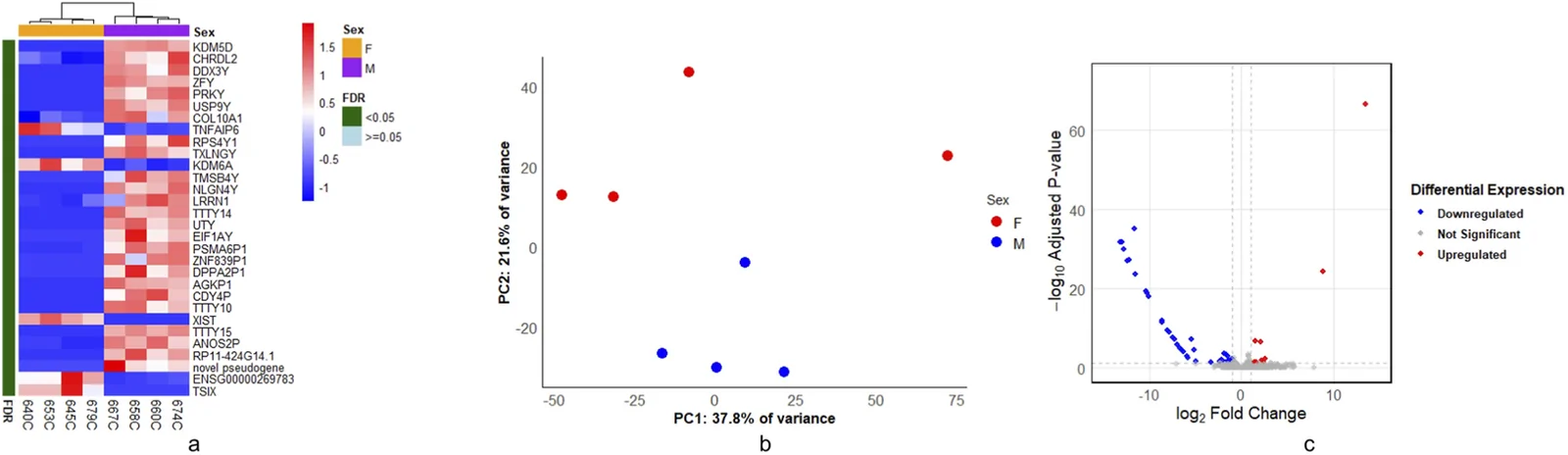
Differential gene expression analysis between male and female osteoarthritis patients. **(a)** Heatmap of the Top 30 differentially expressed genes in local patient cohort. **(b)** Principal Component Analysis between male and female samples. **(c)** Volcano plot of differential gene expression analysis.

Among 20,799 genes with non-zero read counts analyzed, DGE analysis identified 35 upregulated genes (0.17%) and 74 downregulated genes (0.36%) with an adjusted p-value <0.1. The heatmap shows the top 30 differentially expressed genes between male and female patients with OA from our study cohort ranked by adjusted p-values. Samples were clustered based on sex, to illuminate sex-specific expression patterns between male and female patients with OA in our study cohort. In contrast to the GEO data, which showed limited differentially expressed genes, there were 109 differentially expressed genes in our cartilage sample DGE analysis (Figure 3a). Important genes on the heatmap and their roles in OA are presented in Table 3. The PCA analysis performed with the VST data to assess the overall clustering of the samples showed sex-specific clustering in gene expression with PC1 and PC2 accounting for 37.8% and 21.6% of the total variance, respectively (Figure 3b; Supplementary Figures S1,S2). The volcano plot demonstrates significantly upregulated (red) and downregulated (blue) genes (Figure 3c).

**TABLE 3:** Differentially Expressed Genes and their functions.

NCBI link	Genes	Functions	Sex pattern	References
KDM5D lysine demethylase 5D [Homo sapiens (human)] – Gene - NCBI	KDM5D	Located on Y chromosome and it is involved in gene regulation (histone demethylase), cell development, cancer progression, and sex-specific differences in gene expression	Upregulated in Males	​
CHRDL2 chordin like 2 [Homo sapiens (human)] - Gene - NCBI	CHRDL2	A secreted glycoprotein that prevents BMPs from binding to their surface receptors. It may be involved in myoblast and osteoblast differentiation and maturation. It may also inhibit cartilage formation and regeneration	Upregulated in Males	​
ZFY zinc finger protein Y-linked [Homo sapiens (human)] - Gene - NCBI	ZFY	A zinc finger protein involved in male development and spermatogenesis. The specific role in OA remains unclear; however, a study investigating dysfunctional sub-pathways in OA, identified by combining long non-coding RNAs (lncRNA)-mRNA expression profiles, revealed a significant co-expression interaction between LINC00476 and ZFY.	Upregulated in Males	​
PRKY protein kinase Y-linked (pseudogene) [Homo sapiens (human)] - Gene - NCBI	PRKY	Similar to protein kinase and classified as a pseudogene. Hence, it is unlikely to express protein. No direct evidence linking PRKY to OA.	Upregulated in Males	​
USP9Y ubiquitin specific peptidase 9 Y-linked [Homo sapiens (human)] - Gene - NCBI	USP9Y	Located on the Y chromosome and encodes a protein similar to ubiquitin-specific proteases. Essential for spermatogenesis; no role in OA.	Upregulated in Males	​
COL10A1 collagen type X alpha 1 chain [Homo sapiens (human)] - Gene - NCBI	COL10A1	Encodes alpha chain of type X collagen and is expressed by hypertrophic chondrocytes during endochondral ossification and OA progression. Its downregulation seems to have a role in the establishment of a defective and/or unstable subchondral cartilage matrix in OA. Chondrocyte hypertrophy is more pronounced in males, leading to cartilage degeneration and calcification (osteogenic OA phenotype)	Upregulated in males	(Chen et al., 2023; Qian et al., 2024)
TNFAIP6 TNF alpha induced protein 6 [Homo sapiens (human)] - Gene - NCBI	TNFAIP6	Also known as TSG-6. It is a secreted protein that contains hyaluronan-binding LINK domain involved in extracellular matrix stability and cell migration. Its secretion is induced by inflammatory cytokines such as TNF-α and IL-1. It also modulates inflammation by forming a stable complex with inter-alpha-inhibitor (IαI). It inhibits BMP2 dependent differentiation of MSCs into osteoblast. Negative regulation of inflammatory response, positive regulation of cell migration, neutrophil degranulation, cell-cell signaling, mitotic cell cycle. Basically, anti-inflammatory and tissue protective activities. Increased in both cartilage and synovium tissue during OA. It may contribute to OA in diabetic patients through the recruitment of pro-inflammatory CD14 + monocytes in the synovium	Upregulated in Females	(Liu et al., 2024; Moore et al., 2022)
RPS4Y1 ribosomal protein S4 Y-linked 1 [Homo sapiens (human)] - Gene - NCBI	RPS4Y1	Encodes ribosomal protein S4, a component of the 40 S subunit. It may influence male-specific cellular functions in joint tissues, and it is associated with distinct fibrocartilage chondrocytes population	Upregulated in Males	​
TXLNGY taxilin gamma Y-linked (pseudogene) [Homo sapiens (human)] - Gene - NCBI	TXLNGY	A pseudogene	​	​
KDM6A lysine demethylase 6A [Homo sapiens (human)] - Gene - NCBI	KDM6A	Histone Demethylase is located on the X chromosome and is essential for gene regulation during development. KDM6A inhibition can mitigate OA symptoms, including cartilage degradation and bone loss. It mediates joint destruction by epigenetic disturbance of SOX9 promoter and Histone H3K27	Upregulated in Females	(Xu et al., 2022; He et al., 2020; Lian et al., 2023)
TMSB4Y thymosin beta 4 Y-linked [Homo sapiens (human)] - Gene - NCBI	TMSB4Y	Lies within the male specific region chromosome Y. It plays a significant role in wound healing, angiogenesis, immune function, and cancer progression. Tymosin beta-4 X levels in the serum and synovial fluid of Knee OA patients are significantly correlated with disease severity; no such role for TMSB4Y	Upregulated in Males	​
NLGN4Y neuroligin 4 Y-linked [Homo sapiens (human)] - Gene - NCBI	NLGN4Y	Neuroligin family member involved in synaptic functions and neurodevelopmental diseases (ASD). NLGN4Y was significantly downregulated in late-passage male OA chondrocytes and its downregulation might be linked to OA progression	Upregulated in males	​
LRRN1 leucine rich repeat neuronal 1 [Homo sapiens (human)] - Gene - NCBI	LRRN1	Leucine Rich Repeat Neuronal 1 encodes a protein characterized by LRR domains which is involved in protein-protein interactions and neuronal development. In OA, not really significant but has appeared in gene expression analysis between healthy and OA cartilage. May play a role in neuroinflammation relating to pain	Upregulated in males	​
TTTY14 testis expressed transcript, Y-linked 14 [Homo sapiens (human)] - Gene - NCBI	TTTY14	Testis-specific transcript, Y-linked 14. Not disease specific	Upregulated in male	​
UTY ubiquitously transcribed tetratricopeptide repeat containing, Y-linked [Homo sapiens (human)] - Gene - NCBI	UTY	Ubiquitously Transcribe Tetratricopeptide Repeat Containing, Y-Linked, encodes a protein involved in histone demethylation and shares homology with KDM6A. Involved in epigenetic regulation and a marker of male sex	Upregulated in male tissues	​
EIF1AY eukaryotic translation initiation factor 1A Y-linked [Homo sapiens (human)] - Gene - NCBI	EIF1AY	Eukaryotic Translation Initiation Factor 1A, Y-linked. It is also protein coding, and has no link with OA.	Upregulated in males	(Wang et al., 2020)*
PSMA6P1 proteasome subunit alpha 6 pseudogene 1 [Homo sapiens (human)] - Gene - NCBI	PSMA6P1	Proteasome subunit alpha 6 pseudogene 1, does not produce a functional protein	Upregulated in Males	​
ZNF839P1 zinc finger protein 839 pseudogene 1 - NIH Genetic Testing Registry (GTR) - NCBI	ZNF839P1	Zinc Finger Protein 839 Pseudogene 1	Upregulated in Males	​
DPPA2P1 developmental pluripotency associated 2 pseudogene 1 [Homo sapiens (human)] - Gene - NCBI	DPPA2P1	Developmental pluripotency associated with 2 Pseudogene 1. Does not encode functional protein	Upregulated in Males	​
AGKP1 AGK pseudogene 1 [Homo sapiens (human)] - Gene - NCBI	AGKP1	Another pseudogene	Upregulated in males	​
CDY4P chromodomain Y-linked 4 pseudogene [Homo sapiens (human)] - Gene - NCBI	CDY4P	Another nonfunctional pseudogene	Upregulated in males	​
TTTY10 testis expressed transcript, Y-linked 10 [Homo sapiens (human)] - Gene - NCBI	TTTY10	Testis expressed transcript, Y-linked 10	Upregulated in males	​
XIST - Gene - NCBI	XIST	X inactive specific transcript	Upregulated in females	(Cheleschi et al., 2025; Wang et al., 2022)
TTTY15 testis expressed transcript, Y-linked 15 [Homo sapiens (human)] - Gene - NCBI	TTTY15	Testis expressed transcript, Y-linked 15. It has some roles in prostate and lung cancers. LncRNA sponge for microRNAs that modulate gene expression, cell proliferation, and apoptosis	Upregulated in males	(Huang et al., 2023)*
ANOS2P anosmin 2, pseudogene [Homo sapiens (human)] - Gene - NCBI	ANOS2P	Anosmin 2, pseudogene	Upregulated in males	​
DICE Database - RP11-424G14.1	RP11-424G14.1	RP11-424G14.1 is a human-specific, Y-linked long non-coding RNA that regulates immune cell homeostasis in males by supporting IGFBP3 expression and suppressing NF-kB–mediated inflammation. Its loss leads to increased inflammatory signaling, cellular senescence, and metabolic disruption, highlighting its role as a male-specific anti-inflammatory regulator	Upregulated in males	​
TSIX TSIX transcript, XIST antisense RNA [Homo sapiens (human)] - Gene - NCBI	TSIX	Antisense transcript to XIST.	Upregulated in females	(Dong et al., 2024)*

* These studies do not perform sex stratification and thus, their apparent diagnostic value may be confounded by sex-linked expression differences rather than disease-driven regulation.

#### 3.2.2 Functional annotation and pathway enrichment analysis

**FIGURE 4:**
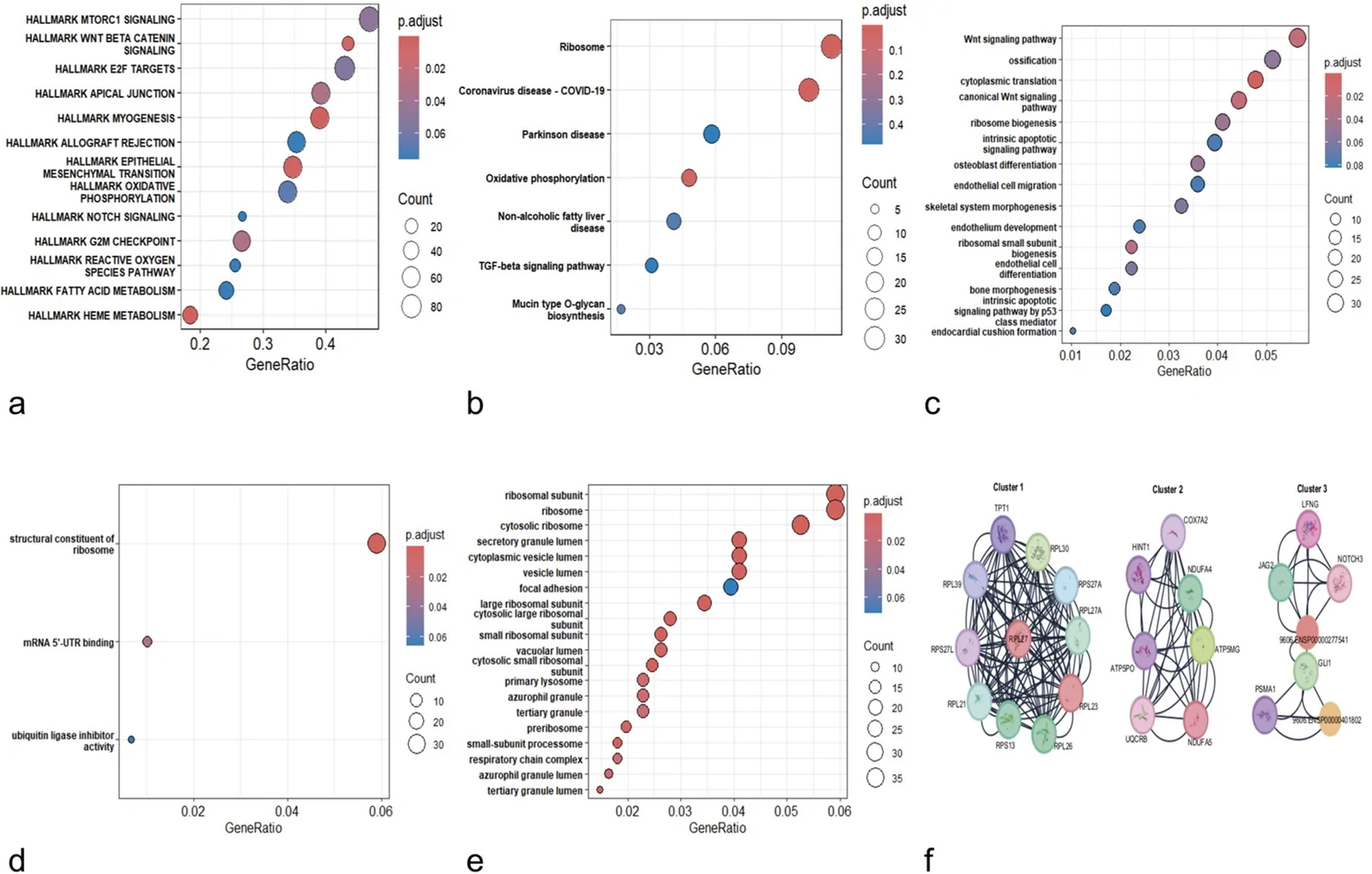
Functional enrichment analysis of DEGs between female and male osteoarthritis patients. **(a)** GSEA identified hallmark pathways, including “MTORC1 signaling”, “oxidative phosphorylation”, and “inflammatory response” **(b)** KEGG pathway analysis revealed significant pathways such as “Ribosome” and “Oxidative phosphorylation” (adjusted p < 0.05), emphasizing metabolic and translational processes; terms are displayed up to an adjusted p < 0.5 for completeness, with significance indicated by the color scale. **(c–e)** GO enrichment analysis identified key biological processes (BP), molecular functions (MF), and cellular components (CC), such as the “Wnt signaling pathway,” “ossification,” “structural constituent of ribosome,” and “cytosolic ribosome,” highlighting the interplay of signaling, structural, and cellular mechanisms **(f)** PPI network analysis revealed three key clusters involving ribosomal and oxidative phosphorylation-related proteins alongside distinct signaling modules such as the NOTCH and Hedgehog pathways.

Similar to the GSE114007 differential gene expression analysis, we conducted KEGG, GSEA, and GO enrichment analyses to explore the possible biological mechanisms underlying sex differences in OA using our local patient cohort. In this analysis, enriched terms significant at adjusted p < 0.05 were considered the primary findings for KEGG and GO. Given the fewer significant KEGG pathways (e.g., Ribosome and Oxidative phosphorylation), the KEGG dot plot (Figure 4b) display threshold was relaxed to 0.5 for completeness. GSEA was used to analyze the enrichment of gene sets across an entire ranked list of genes using log2 fold change. GSEA identified significant enrichment of hallmark pathways related to inflammation, tissue remodeling, metabolic processes, developmental regulation, and cell proliferation and growth in the gene expression data of female patients compared with males with OA within our patient cohort (Figure 4a). Distinct biological pathways involving cellular activities such as inflammation, myogenesis, oxidative phosphorylation are more pronounced in the OA dataset, given that certain signaling pathways including WNT Beta Catenin and mTORC1 signaling suggest potential sex differences (Cheng et al., 2022; Wang Y. et al., 2019; Chen et al., 2025).

KEGG pathway analysis revealed the enrichment of pathways related to protein synthesis (ribosome), metabolism (oxidative phosphorylation), signaling (TGF-beta signaling), and disease-specific processes (COVID-19) (Figure 4b). GO enrichment analysis identified enrichment across pathways such as Wnt signaling, structural constituents of ribosomes, intrinsic apoptotic signaling, cytoplasmic translation, and secretory granules (Figures 4c–e). The PPI network parameters for filtering were similar to those used for the GSE114007 analysis. This analysis revealed three distinct clusters of interacting proteins. Cluster 1 is enriched with ribosomal proteins involved in protein synthesis and translation (e.g., RPL27, RPL23, RPL26, RPL30, RPL21, RPL39, RPS13, RPS27L, RPS27A, RPL27A, and TPT1). Cluster 2 comprises mitochondrial proteins involved in energy metabolism and oxidative phosphorylation (e.g., NDUFA4, NDUFA5, UQCRB, COX7A2, ATP5PO, ATP5MG, and HINT1). Cluster 3 featured key regulators of Notch signaling pathways (e.g., NOTCH3, JAG2, LFNG, GLI1, and PSMA1), suggesting their roles in cell communication and tissue remodeling (Figure 4f).

**FIGURE 5:**
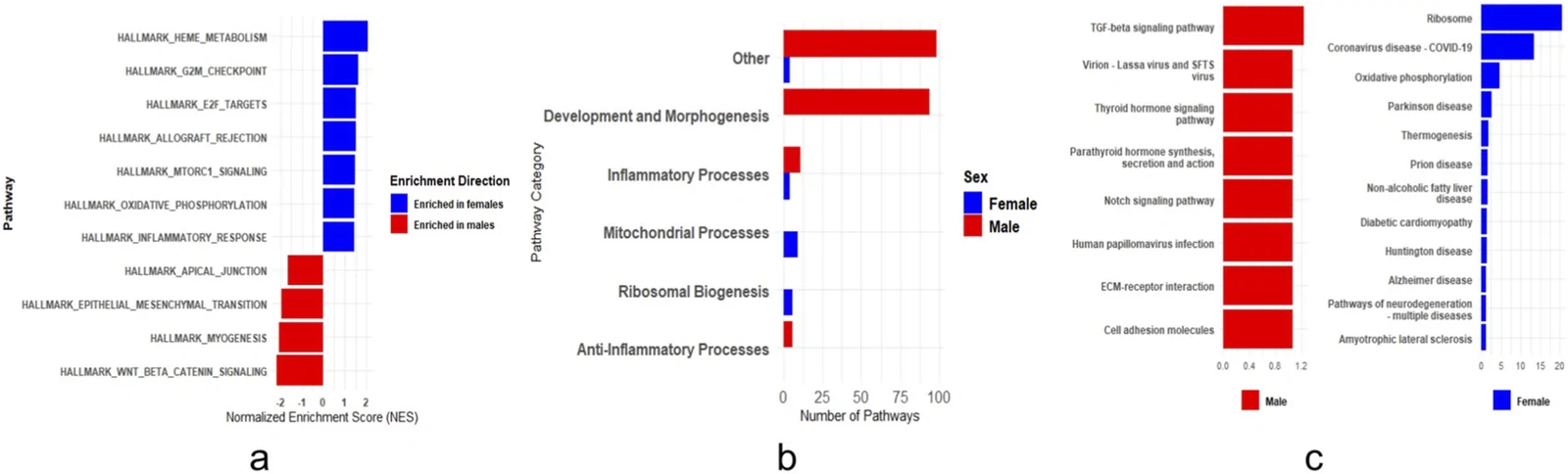
Sex difference analysis using functional enrichment analysis. **(a)** GSEA identified hallmark pathways, including MTORC1 signaling, oxidative phosphorylation, and immune-related processes such as allograft rejection and reactive oxygen species pathway. **(b)** GO analysis showed that females OA samples were enriched in mitochondrial processes and development-related pathways, such as oxidative phosphorylation and cytoplasmic translation, suggesting a broader activation of metabolic and biosynthetic pathways, whereas males had fewer enriched pathways, such as anti-inflammatory processes, inflammatory processes, and development and morphogenesis. **(c)** KEGG pathway analysis also showed similar trends, with female OA patients showing enrichment in oxidative phosphorylation and other metabolic pathways, while males OA samples were enriched in signaling pathways such as Notch and ECM-receptor interactions.

Sex difference analysis of the enrichment pathways revealed distinct sex-specific pathways in OA, with notable differences in inflammation, mitochondrial processes, and tissue remodeling. GSEA analysis revealed significant enrichment in pathways in the female tissue such as oxidative phosphorylation, fatty acid metabolism, and heme metabolism, suggesting increased mitochondrial activity and metabolic dysfunction in female OA patients compared with males. Conversely, males showed greater expression of pathways relating to myogenesis, Wnt signaling in cellular homeostasis, and epithelial-mesenchymal transition (EMT), emphasizing potentially better tissue remodeling and repair processes (Figure 5a). GO analysis in female OA patients revealed an increased number of pathways relating to mitochondrial processes and development, such as oxidative phosphorylation and cytoplasmic translation, whereas male OA patients had increases in pathways relating to inflammatory, anti-inflammatory, and morphological processes including endothelial development and ossification (Figure 5b). KEGG pathway analysis mirrored these trends, with females showing an increase in oxidative phosphorylation and metabolic pathways, while males showed increased signaling pathways such as Notch and ECM-receptor interactions (Figure 5c).

## 4 Discussion

This study analyzed public transcriptomic data from the GEO database and cartilage samples collected from patients who underwent total knee arthroplasty for advanced degenerative OA at our institution. The use of NGS data from both public and local sources allowed us to compare the findings from different sources and elucidate sex-specific differences in gene expression and signaling pathways between males and females with late-stage OA. Surprisingly, the sample obtained from GEO only provided one significant DEG, which was sex related. Despite this shortcoming, we proceeded with pathway enrichment analysis using more relaxed criteria to obtain an overall picture of what to expect with sex differences in patients with OA. The analyses showed that female patients’ transcriptomic data exhibited more inflammatory responses with the upregulation of pathways related to KRAS, NF-kB, and MTORC1 signaling. NF-κB activation in OA cartilage induces TNF-α, IL-6, and MMP-13, which each promote cartilage breakdown (Fernandes et al., 2002; Rigoglou and Papavassiliou, 2013), whereas TNF-α concurrently inhibits cartilage repair, favoring degeneration (Fernandes et al., 2002). The enrichment of Th17-associated cytokine signaling in our dataset also supports prior findings that IL-17–driven inflammation accelerates cartilage catabolism and chondrocyte apoptosis (Liu et al., 2022; Yang et al., 2019; Yang and Wang, 2015). Other pathway enrichment and PPI analyses have also revealed an interesting dichotomy related to immunological differences, ECM organization, cell cycle regulation, apoptosis, chondrocyte proliferation, and cartilage remodeling (Liu et al., 2022; Yang and Wang, 2015). These findings suggest that sex-specific differences in inflammation, ECM dynamics, and cell cycle dysregulation may drive OA progression.

The findings from our tissue cohort showed a much clearer picture of sex differences at the transcriptomic level. Differences in the upregulation of genes such as COL10A1, TNFAIP6, and KDM6A provide significant insights. The upregulation of COL10A1 in male samples is a hallmark of chondrocyte hypertrophy that may reflect greater mechanical stress or structural joint damage (Chen et al., 2023; Qian et al., 2024). In contrast, the upregulation of TNFAIP6, a TNF-α-inducible protein that stabilizes the ECM, whose secretion is induced by inflammatory cytokines such as TNF-α and IL-1, and KDM6A in females, indicates an ongoing inflammatory process and cartilage degradation (Liu et al., 2024; Moore et al., 2022). Functional enrichment analyses showed significant immune (Allograft Rejection, Reactive Oxygen Species), metabolic (mTORC1, Oxidative Phosphorylation, Fatty Acid, Heme, and protein synthesis), and signaling/cell cycle pathways in females. These processes align with prior studies that showed that oxidative phosphorylation, ribosomal activity, and Wnt signaling contribute to OA pathophysiology through altered chondrocyte metabolism and matrix turnover (Cheng et al., 2022; Wang Y. et al., 2019; Dudaric et al., 2023; van den Akker et al., 2022). Males were enriched for pathways related to development (Notch and TGF-β signaling) and structural remodeling (Wnt/β-catenin signaling, myogenesis, and epithelial–mesenchymal transition), which are pathways previously implicated in cartilage differentiation and repair (Saito and Tanaka, 2017; Smith et al., 2023). Together, these results suggest that males predominantly experience structural and mechanical drivers of OA progression, whereas females exhibit a stronger inflammatory and metabolic signature.

Several bioinformatics studies have explored sex differences in osteoarthritis. Yang et al. performed a sex difference analysis based on three data sets obtained from GEO database (Yang et al., 2020). Their study agrees on several levels with our findings, as they concluded that OA is sex-dependent at the transcriptomic level. Moreover, their analysis revealed that females exhibited more ribosomal/protein synthesis and ECM/immune-related processes, with hub genes such as EEF2, EEF1A1, RPL37A, and FN1 being highly expressed in female samples. In contrast, males showed stress response- and apoptosis-related signatures, with hub genes such as BCL2L1, HNRNPD, and PABPN1 being downregulated (Yang et al., 2020). These findings align with our analyses, despite the use of microarray data. However, in our data, males also exhibited enrichment of ECM interaction, Wnt/β -catenin, and TGF- β signaling, pointing more toward structural remodeling as a key driver in male OA pathogenesis. Furthermore, our analysis identified sex differences in some genes with functional relevance. KDM6A, a histone demethylase that activates RUNX2 to regulate chondrocyte differentiation and exhibits a protective effect in knockdown murine models (He et al., 2020; Liao et al., 2017; Lian et al., 2023), was upregulated in our female samples, which is consistent with prior reports (Xu et al., 2022). XIST, a long non-coding RNA upregulated in females, has been shown to promote OA progression by increasing chondrocyte apoptosis and monocyte adhesion (Cheleschi et al., 2025; Wang et al., 2022). Similarly in a study looking at TSIX, the antisense transcript of XIST, it was found to be upregulated in serum samples of OA patients compared to healthy patients (Dong et al., 2024). However, this study did not stratify the results based on sex, thus it is unclear whether the differences in levels of TSIX were driven by female sex. Given that TSIX is an X-linked, XIST-regulating IncRNA typically active in females, it is biologically plausible that female sex accounted for much of the observed difference. Our findings, showing TSIX upregulation exclusively in female OA cartilage may therefore provide a more refined, tissue-specific explanation of the same trend observed systemically in serum.

Li and Zheng analyzed a published transcriptome dataset to gain insight into the relationship between sex and OA (Li and Zheng, 2021). They established molecular sex differences between healthy and OA cohorts by comparing a sex-specific healthy cohort with their OA counterparts, which is consistent with our analysis. Furthermore, Li and Zheng’s sex-dependent analyses revealed that ECM remodeling and immune signaling (IL4/IL-13, RUNX3, and FOXO) drive OA pathogenesis in the female cohort, whereas ER stress, apoptosis, and NGF signaling drive OA development in the male cohort (Li and Zheng, 2021). Their study positioned ECM-related changes more in females, while our study emphasized these changes in both sexes, with males exhibiting remodeling and females exhibiting inflammatory/immune-linked changes. While the pathways identified may differ from our findings, the overall conclusion is similar, with females showing stronger immune/inflammatory-driven mechanisms and males showing stress- and remodeling-driven mechanisms. It is worthwhile pointing out that our analysis integrated both public and local cohorts, thus providing more translational significance.

Wang et al. compared microarray data of synovial membrane samples from postmenopausal females with OA to males with OA and identified hub genes such as EGF, ERBB2, CDC42, and STAT1 (Wang S. et al., 2019). Their enrichment analyses highlighted PI3K-Akt signaling, osteoclast differentiation, and focal adhesion, positioning immune and ECM-related processes more in females, supporting our findings (Wang S. et al., 2019). However, our study also emphasized remodeling programs in males from both public and local cohorts in advanced-stage cartilage tissues, which further support the sex-driven pathophysiology in OA development. Atasoy-Zeybek et al.‘s recent review article on the impact of aging and estrogen in OA describes how estrogen deficiency and aging synergistically drive OA pathogenesis through inflammation, oxidative stress, and ECM degradation (Atasoy-Zeybek et al., 2025). They highlighted that postmenopausal estrogen loss increases IL-1β, IL-6, TNF-α, MMP-13, and ADAMTS expression in cartilage and linked estrogen receptor signaling (ERα/ERRγ) to chondrocyte senescence, mitochondrial dysfunction, and loss of proteostasis. Specifically, ERRγ activation promotes catabolic signaling via the IL-6–MAPK/ERK pathway (Atasoy-Zeybek et al., 2025). The findings of this review align with our observed female-specific enrichment in immune and metabolic pathways.

While our findings highlight a clear sex-dependent dichotomy in human OA cartilage, other studies have reported contrasting patterns in different biological contexts. It is important to note that most mouse OA studies use surgical models in young mice, which may not reflect the age-related changes seen in the context of human OA (Atasoy-Zeybek et al., 2025; Iijima et al., 2022). Bergman et al. used a post-traumatic OA mouse model to demonstrate that males exhibited greater joint damage, more severe synovitis, and higher MMP activity, while their study’s synovial transcriptomics analysis showed sustained activation of inflammatory, fibrotic, and angiogenic pathways in males compared to females (Bergman et al., 2024). Their study suggested that injury-driven inflammation may persist more strongly in males than in females (Bergman et al., 2024). Furthermore, another study that characterized the structural and proteomic changes associated with knee OA in mice across the lifespan and by sex showed that aging-induced cartilage degeneration is more peculiar to male mice than to female mice (Iijima et al., 2023). This finding opposes the findings of human OA studies (Atasoy-Zeybek et al., 2025). Although not observed in our study, other gene expression analyses of sex differences in OA have reported several hub genes with potent roles in various biological processes involved in OA pathogenesis (Xue et al., 2023; Yang et al., 2020; Atasoy-Zeybek et al., 2025; Li and Zheng, 2020). Feng et al. examined the Gene Expression Omnibus database and found that the expression of genes encoding Ca2+/calmodulin-dependent protein kinase-4 (CaMK4) was significantly higher in female patients with OA than in male and healthy female controls (Feng et al., 2023). Their study also demonstrated that CaMK4 inhibition in a DMM mouse model reduced cartilage damage and inflammation, highlighting CaMK4 as a potential therapeutic target for OA (Feng et al., 2023). However, grouping results by sex remains challenging because of the variability and complexity of the data and findings.

This study has some limitations. First, our local patient cohort of eight patients was relatively small due to various reasons, including strict inclusion and exclusion criteria and challenges with processing cartilage tissue. Most of our patients have grade IV OA, making it challenging to obtain adequate tissue samples for RNA extraction. Our study criteria excluded patients with comorbidities such as diabetes, cancer, and any form of immunosuppressive disease; those with a current smoking history; and those aged 75 years or older. In our analysis, subject-level variables such as age and BMI were controlled with no statistical effect on the analysis. However, other variables such as medication use and anatomical alignment were not systematically controlled for in the transcriptomic analyses. These factors may contribute to gene expression variability and represent important considerations for future studies with larger, better-characterized cohorts. In addition, our analysis was limited to the articular cartilage; as OA is a whole-joint disease, incorporating synovium, synovial fluid, and meniscal tissue will be needed to fully characterize sex-specific differences across the knee joint in future studies. As our analysis was correlative, the genes and pathways identified should be regarded as hypothesis-generating, and future experimental validation will be needed to confirm their functional roles in OA development and progression. Therefore, while our conclusions may not be fully generalizable, the integration of transcriptomic data from the GEO database strengthened the credibility and reproducibility of our findings. One particular strength of this study is the use of RNA sequencing data instead of microarray data, as seen in many similar studies. While microarray is still an effective technology for discovering DEGs, RNA sequencing has been shown to be more precise, making it a more effective technology for discovering low-abundance genes and for more accurate quantification of the expressed genes present in a sample (Zhao et al., 2014). RNA sequencing is also known for its high speed and throughput, as well as its greater overall specificity (Negi et al., 2022). In addition, the use of NGS data can provide more detailed gene coverage and quantification, especially for lowly abundant genes, which may provide deeper insights into the sex differences observed in OA pathogenesis. We believe that these findings will contribute to the growing evidence of sex-dependent mechanisms underlying OA pathogenesis.

## 5 Conclusion

This study identified several genes and pathways that may explain the differences in OA pathogenesis between males and females. The broader picture seen from our findings suggests that while both sexes share core OA pathobiological processes, the predominant mechanisms may differ by sex. OA in females appears to be characterized by a greater relative contribution of inflammatory and metabolic mechanisms, whereas OA in males appears to be driven more prominently by structural pathways involved in stress and remodeling. These findings do not preclude the presence of inflammatory processes in males or structural remodeling in females, but rather highlight sex-specific differences in the relative predominance of these mechanisms. While our study does not infer causality, it provides additional insight into a disease process with multifactorial etiologies and complex pathophysiology. This study reinforces a growing paradigm shift toward sex-based therapeutic research in OA. Although genetic factors contribute significantly to OA pathogenesis, epigenetic, anatomical, and environmental influences may play some role in the observed sex differences. Therefore, more studies are needed to better understand these sex differences and to design effective therapeutics that can help patients within the treatment gap who cannot undergo surgery due to different medical, economic, or cultural barriers (Faust and Dy, 2024; Mandalia and Shah, 2024; Salazar et al., 2019). Looking forward, further defining patients by clinical phenotype and integrating multi-omics data (transcriptomics, proteomics, metabolomics, and genomics) may offer a more robust strategy for identifying subgroups and advancing targeted OA therapeutics.

## Data Availability

The datasets presented in this study can be found in online repositories. The names of the repository/repositories and accession number(s) can be found below: Publicly available dataset GSE114007 was analyzed in this study and can be found in the 717 NCBI Gene Expression Omnibus (GEO) repository 718 (https://www.ncbi.nlm.nih.gov/geo/query/acc.cgi?acc=GSE114007).
